# Protective Effect of Edaravone on Doxorubicin-Induced Thyroid Dysfunction in Rats Revealed by ^99m^Tc Pertechnetate Thyroid Gland Scintigraphy and Biochemical Methods

**DOI:** 10.3390/medicina62050894

**Published:** 2026-05-06

**Authors:** Murat Kalın, Hatice Aygun, Haluk Kerim Karakullukcu, Mina Karakullukcu, Aylin Arslan, Serdar Savas Gul, Ömer Faruk Özkan, Gülçin Ercan

**Affiliations:** 1 Department of General Surgery, Sultan 2. Abdulhamid Han Educational and Research Hospital, University of Health Sciences, Istanbul 34668, Türkiye; murat.kalin@hotmail.com (M.K.); halukkarakullukcu@gmail.com (H.K.K.); omerfaruk.ozkan@sbu.edu.tr (Ö.F.Ö.); 2 Neuroscience Laboratory, Biruni University Research Center (BAMER), Biruni University, Istanbul 34010, Türkiye; hatice_5aygun@hotmail.com; 3 Department of Oncology, Sultan 2. Abdulhamid Han Educational and Research Hospital, University of Health Sciences, Istanbul 34668, Türkiye; mina.zoralioglu@gmail.com; 4 Department of General Surgery, Istanbul Prof. Dr. Cemil Taşçıoğlu City Hospital, University of Health Sciences, Istanbul 34668, Türkiye; aylinarslan53@outlook.com; 5 Department of Nuclear Medicine, Faculty of Medicine, Lokman Hekim University, Ankara 06530, Türkiye; serdar.gul@lokmanhekim.edu.tr

**Keywords:** doxorubicin, hypothyroidism, edaravone, ^99m^Tc pertechnetate, thyroid scintigraphy, thyroid hormones

## Abstract

*Background and Objectives*: Doxorubicin is an antineoplastic drug used to treat cancer. However, side effects limit its use. Edaravone (EDO) is a recently discovered, powerful drug with antioxidant properties. The aim of the present study was to show the negative effects of doxorubicin and the protective effect of EDO on the thyroid gland using scintigraphic and biochemical methods. *Materials and Methods*: Thirty-five male Wistar rats were randomly divided into five groups (n = 7) to establish the following study groups: control, doxorubicin, and 1, 10, or 30 mg/kg EDO. DOX (18 mg/kg cumulative intraperitoneal injection (i.p.) was performed on the 19th, 20th, and 21st days of the experiment. EDO (1, 10, and 30 mg/kg) was administered on the first day of the trial and continued for 21 days. These groups also received i.p. injections of DOX (18 mg/kg) on the 19th, 20th, and 21st days of the experiment. On the 22nd day of the experiment, scintigraphic imaging of the thyroid glands of rats was performed using ^99m^Tc pertechnetate as the radiopharmaceutical. Serum levels of T3, T4, TSH, NLRP3, IL-1β, and IL-18, as well as thyroid tissue levels of MDA, TNF-α, and IL-6, were determined using the ELISA method. *Results*: DOX significantly reduced ^99m^Tc pertechnetate uptake in the thyroid gland compared to the control group (*p* < 0.001). It reduced plasma levels of thyroid hormones T3 (*p* < 0.001) and T4 (*p* < 0.001) while increasing TSH levels (*p* < 0.01). Additionally, NLRP3, IL-1β, IL-18, MDA, TNF-α, and IL-6 levels were significantly increased in the DOX group compared with the control group (all *p* < 0.001). Pretreatment with EDO significantly attenuated doxorubicin-induced abnormalities in the thyroid gland (*p* < 0.001). *Conclusions*: The data from scintigraphic and biochemical analyses revealed the development of hypothyroidism after doxorubicin administration in rats. It was shown that pretreatment with EDO could partially prevent hypothyroidism caused by doxorubicin.

## 1. Introduction

Doxorubicin (DOX) is a broad-spectrum antineoplastic cancer drug used to treat lung, ovary, breast, uterus, lymphoma, and leukemia cancers [1]. It is well established that the rate of remission increases significantly and that survival time is prolonged in anticancer treatments with DOX. Although this chemotherapeutic agent is highly effective, its use is limited by its side effects [2,3].

The body’s largest hormone-producing endocrine gland is the thyroid gland, and it plays an important role in almost all cellular functions. The hormones triiodothyronine (T3) and thyroxine (T4) secreted from the thyroid gland are essential for normal growth and development in all tissues in the body and are important regulators of metabolism [4,5]. Past and recent studies have shown that chemotherapy drugs affect thyroid gland function. Thyroid hypofunction is especially common after radiotherapy and chemotherapy [6,7,8]. For this reason, agents to prevent thyroid dysfunction caused by chemotherapeutic agents are especially important. Although a small number of experimental studies have reported doxorubicin-induced alterations in thyroid hormone levels and hypothyroid-like effects [9,10,11], evidence directly addressing the functional status and activity of the thyroid gland remains limited.

Edaravone (EDO) is a newly discovered, powerful antioxidant drug. EDO (3-methyl-1-phenyl-2-pyrazoline-5-one) has been recently introduced for the treatment of adult acute ischemic brain disorders and amyotrophic lateral sclerosis (ALS) [12]. In many studies, EDO has demonstrated cell-protective properties by mitigating the side effects of chemotherapeutic agents [13,14]. In experimental studies, EDO has been shown to reduce neurodevelopmental changes caused by cisplatin and DOX, inhibit autotoxicity, reduce renal dysfunction and renal tubular damage, and prevent cardiotoxicity [15,16,17,18]. Recent experimental studies have demonstrated that EDO alleviates thyroid dysfunction by reducing oxidative stress and inflammatory responses in both hypothyroid and hyperthyroid models, primarily by modulating ROS generation and inflammation-associated signaling pathways [19,20]. However, to the best of our knowledge, no previous study has directly investigated the effectiveness of EDO in doxorubicin-induced hypothyroidism, highlighting a gap in the existing literature.

Thyroid scintigraphy provides functional information on thyroid gland activity by visualizing radionuclide uptake; changes in uptake intensity reflect alterations in thyroid hormone synthesis and glandular function, thereby allowing assessment of functional thyroid impairment. Accordingly, thyroid scintigraphy has been increasingly utilized in experimental animal models to evaluate functional alterations of the thyroid gland under various pathological conditions [21,22].

Despite increasing evidence that doxorubicin may affect thyroid hormone homeostasis, studies directly evaluating functional thyroid activity in experimental models remain limited. In particular, functional imaging approaches such as thyroid scintigraphy have rarely been applied to investigate chemotherapy-associated thyroid dysfunction. Therefore, the present study aimed to investigate doxorubicin-associated thyroid dysfunction and to evaluate the potential protective effects of edaravone using both biochemical markers and functional thyroid scintigraphy in an experimental rat model. We hypothesized that pretreatment with edaravone would attenuate doxorubicin-associated thyroid functional impairment.

## 2. Material and Method

### 2.1. Animals

Thirty-five adult male Wistar albino rats (weighing 230–250 g) were included in the present study. The animals were maintained under controlled laboratory conditions, with a constant ambient temperature of 22 ± 2 °C, relative humidity of approximately 50%, and a 12 h light/12 h dark cycle (lights on at 07:00 a.m.).

Rats were housed in standard polypropylene cages equipped with wire-mesh tops. Cage hygiene was ensured by routine cleaning at three-day intervals, and both food and water supplies were refreshed regularly. Throughout the acclimatization period and the experimental phase, animals were allowed ad libitum access to standard laboratory chow and tap water. A 10-day acclimatization period was provided prior to the initiation of experimental procedures.

All experimental protocols were reviewed and approved by the Local Animal Ethics Committee (Approval No: HADYEK-2020/15) and were conducted in accordance with established guidelines for the care and use of laboratory animals.

### 2.2. Experimental Procedures

**Figure 1:**
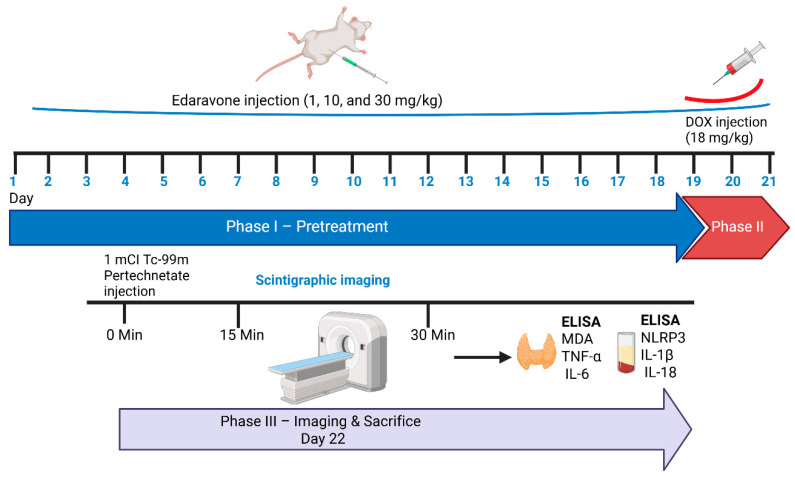
This summarizes the experimental protocol and timeline for EDO pretreatment, doxorubicin administration, scintigraphic imaging, and biochemical analyses. Phase I (Pretreatment phase) consisted of daily intraperitoneal administration of EDO (1, 10, or 30 mg/kg) for 21 consecutive days. In the doxorubicin (DOX) group, rats received physiological saline during this period. Phase II (DOX administration) involved intraperitoneal injection of doxorubicin at a cumulative dose of 18 mg/kg over three consecutive days (days 19–21). Phase III (Imaging and sacrifice) was conducted on day 22. Rats received an intravenous injection of 1 mCi ^99m^Tc pertechnetate, followed by scintigraphic imaging at defined time points (15–30 min post-injection) to evaluate thyroid functional uptake. After imaging, blood samples were collected, animals were euthanized, and thyroid tissues were excised for biochemical analyses. Serum and tissue levels of MDA, TNF-α, IL-6, NLRP3, IL-1β, and IL-18 were subsequently measured using ELISA.

Following the acclimatization period, rats in the treatment groups received edaravone (1, 10, or 30 mg/kg, intraperitoneally) once daily for 21 consecutive days. Subsequently, doxorubicin (18 mg/kg cumulative, intraperitoneally) was administered on three consecutive days. At the end of the experimental protocol, blood samples were collected under anesthesia, after which the animals were sacrificed by euthanasia, and the thyroid glands were rapidly excised for biochemical analyses (Figure 1).

The rats were randomly allocated into five experimental groups, with seven animals per group (n = 7), as outlined below:

Group I: Control group (2 mL/kg saline, intraperitoneal [i.p]).

Group II: Doxorubicin group (18 mg/kg cumulative, i.p., over three consecutive days).

Group III: 1 mg edaravone group (1 mg/kg, i.p. for 21 days) + doxorubicin (18 mg/kg cumulative, i.p. for three days).

Group IV: 10 mg edaravone group (10 mg/kg, i.p. for 21 days) + doxorubicin (18 mg/kg cumulative, i.p. for three days).

Group V: 30 mg edaravone group (30 mg/kg, i.p. for 21 days) + doxorubicin (18 mg/kg cumulative, i.p. for three days).

### 2.3. Drugs

EDO was purchased from Sigma-Aldrich (St Louis, MO, USA) and dissolved in saline. DOX (10 mg/ampule) was purchased at a local pharmacy. Doses of EDO and DOX were determined according to previous studies [14,23,24].

### 2.4. Administration of Drugs

All procedures applied to the rats were performed daily between 09:00 and 10:00, maintaining diurnal rhythm.

Following the acclimatization period, Saline (1 mL/kg, i.p.) was given to the control group for 21 days. In the DOX group, rats did not receive any active treatment during the first 18 days of the experimental period and were administered physiological saline (1 mL/kg, intraperitoneally) once daily during this phase. DOX (cumulative dose of 18 mg/kg, i.p.) was injected in the last three days of the experiment (on the 19–21 days). EDO was administered at the same time every day for 21 days in specified doses (1, 10, and 30 mg/kg).

On the 22nd day of the trial, scintigraphic imaging, withdrawal of heart blood, and euthanasia were performed in rats, and the experiment was terminated.

### 2.5. Scintigraphic Imaging

On the 21st day of the experiment, scintigraphic imaging was performed to evaluate thyroid function. Rats received an intravenous injection of 0.3 mL containing 1 mCi of ^99m^Tc-pertechnetate via the tail vein. Five minutes after tracer injection, animals were anesthetized with ketamine/xylazine (75/10 mg/kg). Scintigraphic acquisition was initiated 15 min after tracer administration.

Rats were positioned in the supine position on the imaging table of a dual-head gamma camera (256 × 256 matrix; Siemens Symbia, Hoffman Estates, IL, USA) equipped with a parallel-hole collimator. Imaging was performed until 300 kilocounts of gamma photons were acquired. As reported in previous studies [25,26], the entire acquisition process was completed in approximately five minutes.

Semi-quantitative evaluation of thyroid radiotracer uptake was performed using scintigraphic images obtained from each animal. A region of interest (ROI) encompassing the thyroid gland was manually delineated on each image. Care was taken to include the entire thyroid uptake area while avoiding adjacent tissues to ensure accurate quantification of thyroid radiotracer activity.

To normalize thyroid uptake values and minimize background interference, a background ROI was placed in the supraclavicular region where no thyroid activity was present. Thyroid uptake was expressed as a target-to-background (T/B) ratio, calculated by dividing the total counts within the thyroid ROI by the counts measured in the background ROI [27,28].

Scintigraphic images were post-processed and analyzed using syngo.via MI Applications software (Siemens Healthineers, Erlangen, Germany). ROI placement and quantitative measurements were performed independently by two experienced nuclear medicine specialists blinded to the experimental groups, and the average of the two measurements was used for statistical analysis. Interobserver reliability for thyroid T/B measurements was good (ICC = 0.85, 95% CI: 0.76–0.92, *p* < 0.001; two-way random-effects model, absolute agreement), indicating a high level of agreement between observers. All scintigraphic acquisitions were performed using the same imaging protocol and acquisition parameters to ensure consistency between measurements.

### 2.6. Taking Blood Samples and Measuring Thyroid Hormones

At the end of the experiment, blood was collected from the hearts of anesthetized rats for biochemical analysis. Then, the blood was transferred to polyethylene tubes containing heparin for analysis of T3, T4, and TSH levels. Tubes were centrifuged at +4 °C at 3000 RPM for 10 min, and plasma was obtained. The plasma samples were stored at −20 °C until being analyzed. T3, T4, and TSH values were measured by ELISA using commercial kits according to the manufacturer’s instructions.

Serum levels of IL-1β, IL-18, and NLRP3 were quantified using commercially available sandwich ELISA kits according to the manufacturers’ instructions.

### 2.7. Biochemical Analysis of Thyroid Tissue

All thyroid tissue samples were rinsed with cold isotonic saline (0.9%). The tissues were then finely minced and homogenized in cold phosphate-buffered saline (PBS, pH 7.4) using stainless steel beads. Homogenates were centrifuged at 2500 rpm for 20 min to obtain supernatants. TNF-α, IL-6, and MDA levels were quantified using commercially available quantitative sandwich ELISA kits (Bioassay Technology Laboratory, Shanghai, China) according to the manufacturer’s instructions. Results were normalized to wet tissue weight and expressed as ng/L for TNF-α and IL-6 and µmol/L for MDA.

### 2.8. Statistical Analyses

Statistical analyses were performed using SPSS Statistics (version 20.0) and GraphPad Prism (version 7.0). Data distribution was assessed using the Kolmogorov–Smirnov test. Comparisons among groups were performed using one-way analysis of variance (ANOVA). When a significant overall effect was detected, Tukey’s post hoc test was applied for pairwise comparisons to control the family-wise error rate.

To further evaluate the potential impact of multiple testing, Bonferroni-adjusted comparisons were additionally performed as a sensitivity analysis, which yielded the same pattern of statistical significance. Data are presented as mean ± standard error of the mean (mean ± SEM). A value of *p* < 0.05 was considered statistically significant.

## 3. Results

### 3.1. Biochemical Assay

**Figure 2:**
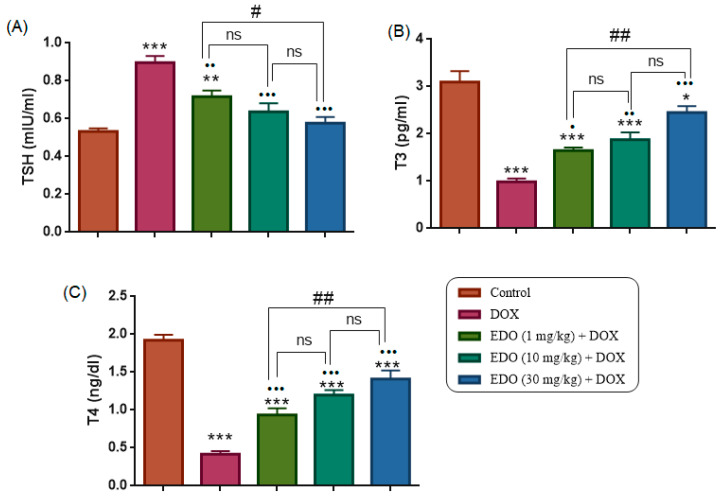
Plasma (**A**) TSH, (**B**) T3, (**C**) T4, and levels of five study groups. Data are presented as mean ± SEM. DOX group has significantly lower plasma T3, T4, and TSH compared to the control group (* *p* < 0.05, ** *p* < 0.01, *** *p* < 0.001). On the other hand, 1, 10, and 30 mg/kg EDO + DOX groups had higher T3 and T4 levels compared to the DOX group (• *p* < 0.05; •• *p* < 0.01; ••• *p* < 0.001). Comparisons among EDO-treated groups showed dose-dependent differences (# *p* < 0.05, ## *p* < 0.01), while ns indicates non-significant differences.

As shown in Figure 2B,C, the DOX group had significantly lower serum T3 and T4 levels compared to the control group (one-way ANOVA: T3, F(4,30) = 31.38, *p* < 0.0001; T4, F(4,30) = 42.93, *p* < 0.0001; Control vs. DOX, T3 *p* < 0.0001, T4 *p* < 0.0001). In addition, serum T3 and T4 levels of the 1, 10, and 30 mg/kg EDO + DOX groups were significantly lower compared to the control group (T3: *p* < 0.0001, *p* < 0.0001, *p* = 0.0246; T4: *p* < 0.0001, *p* < 0.0001, *p* = 0.0288, respectively).

On the other hand, the 1, 10, and 30 mg/kg EDO + DOX groups exhibited significantly higher serum T3 and T4 levels compared to the DOX group (T3: *p* = 0.0209, *p* = 0.0012, *p* < 0.0001; T4: *p* = 0.0293, *p* < 0.0001, *p* < 0.0001, respectively). Within the EDO-treated groups, serum T3 and T4 levels did not differ significantly between the 1 and 10 mg/kg doses (*p* = 0.7996 and *p* = 0.2235, respectively), but were significantly lower at 30 mg/kg compared with the 1 mg/kg dose (*p* = 0.0037 and *p* = 0.0150, respectively).

Serum TSH level was significantly higher in the DOX group compared to the control group (one-way ANOVA: F(4,30) = 18.65, *p* < 0.0001: Control vs. DOX, *p* < 0.0001), indicating the development of a hypothyroid state following doxorubicin administration. Conversely, the 1, 10, and 30 mg/kg EDO + DOX groups showed significantly lower serum TSH levels compared to the DOX group (*p* = 0.0051, *p* < 0.0001, and *p* < 0.0001, respectively). Serum T3, T4, and TSH levels of all groups are shown in Figure 2A.

These findings demonstrate that doxorubicin induces a clear hypothyroid profile, characterized by elevated TSH and reduced T3 and T4 levels, whereas EDO pretreatment effectively mitigates DOX-induced thyroid dysfunction, particularly at higher doses. To further determine whether these biochemical alterations were accompanied by functional changes in thyroid activity, scintigraphic evaluation of thyroid radionuclide uptake was subsequently performed.

### 3.2. Scintigraphic Assay

**Figure 3:**
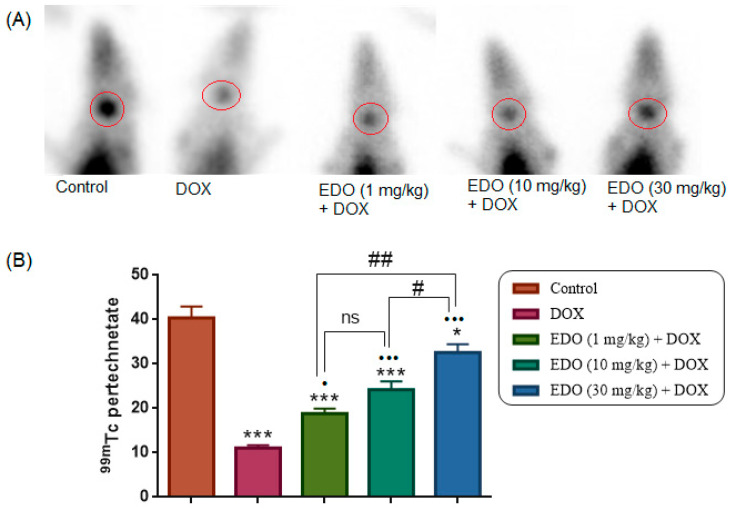
Representative scintigraphic images (**A**) and quantitative analysis (**B**) of ^99m^Tc-pertechnetate thyroid uptake across all study groups. Data are presented as mean ± SEM. The DOX group exhibited significantly lower ^99m^Tc-pertechnetate uptake compared with the control group. Similarly, the 1 and 10 mg/kg EDO + DOX groups showed significantly reduced uptake compared with the control group (* *p* < 0.05, *** *p* < 0.001). In contrast, thyroid ^99m^Tc-pertechnetate uptake was significantly higher in the 1, 10, and 30 mg/kg EDO + DOX groups compared with the DOX group (• *p* < 0.05, ••• *p* < 0.001). Comparisons among EDO-treated groups demonstrated dose-dependent differences, with significant differences at # *p* < 0.05 and ## *p* < 0.01, while ns indicates non-significant comparisons. Red circles indicate regions of interest (ROIs) used for quantitative analysis.

As illustrated in Figure 3A,B, the DOX group had significantly lower ^99m^Tc-pertechnetate uptake levels in the thyroid gland compared to the control group (one-way ANOVA, F(4,30) = 42.93, *p* < 0.0001, Control vs. DOX, *p* < 0.0001). However, ^99m^Tc-pertechnetate uptake in the thyroid gland was significantly reduced in the 1, 10, and 30 mg/kg EDO + DOX groups compared to the control group (*p* < 0.0001, *p* < 0.0001, and *p* = 0.0288, respectively).

**Figure 4:**
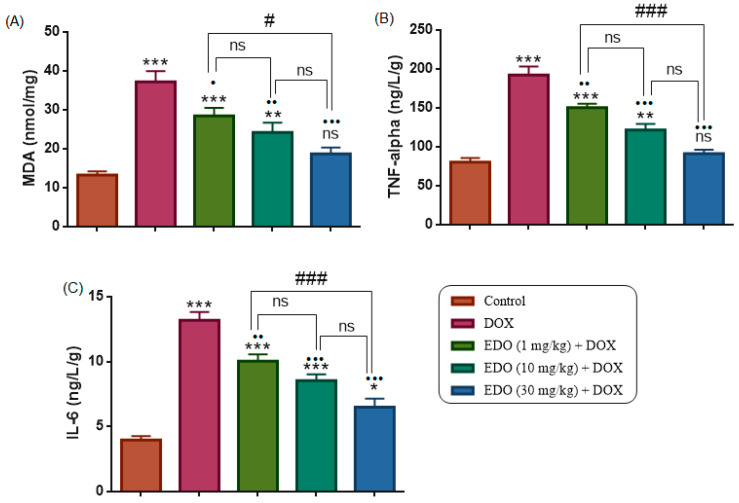
Effects of treatments on (**A**) MDA, (**B**) TNF-α, and (**C**) IL-6 levels. Data are presented as mean ± SEM. * *p* < 0.05, ** *p* < 0.01, *** *p* < 0.001 indicates comparisons with the control group; • *p* < 0.05, •• *p* < 0.01, ••• *p* < 0.001 indicates comparisons with the DOX group; # *p* < 0.05, ### *p* < 0.001 indicates comparisons among EDO-treated groups. ns denotes non-significant differences.

^99m^Tc-pertechnetate uptake levels in the thyroid gland were significantly higher in the 1, 10, and 30 mg/kg EDO + DOX groups compared to the DOX group (*p* = 0.0293, *p* < 0.0001, and *p* < 0.0001, respectively). Among the EDO-treated groups, thyroid ^99m^Tc-pertechnetate uptake differed in a dose-dependent manner, with no significant difference between the 1 and 10 mg/kg EDO + DOX groups (*p* = 0.2235), whereas uptake was significantly lower in the 30 mg/kg EDO + DOX group compared with both the 1 mg/kg (*p* < 0.0001) and 10 mg/kg (*p* = 0.0150) EDO + DOX groups. The ^99m^Tc-pertechnetate uptake levels of the study groups are given in Figure 3, and scintigraphic images of all groups are shown in Figure 4. Following the functional scintigraphic assessment, tissue oxidative stress was evaluated to investigate potential mechanisms underlying the observed thyroid dysfunction.

### 3.3. Tissue MDA Assay

Tissue MDA levels differed significantly among the study groups (one-way ANOVA, F(4,30) = 19.14, *p* < 0.0001). Compared with the control group, the DOX group exhibited significantly higher MDA levels (*p* < 0.0001), while MDA levels were also significantly higher in the 1 and 10 mg/kg EDO + DOX groups (*p* = 0.0002 and *p* = 0.0075, respectively), with no significant difference observed between the control and 30 mg/kg EDO + DOX groups (*p* = 0.3759).

Compared with the DOX group, MDA levels were significantly reduced in the 1, 10, and 30 mg/kg EDO + DOX groups (*p* = 0.0436, *p* = 0.0011, and *p* < 0.0001, respectively).

Within the EDO-treated groups, no significant difference in MDA levels was observed between the 1 and 10 mg/kg EDO + DOX groups (*p* = 0.6150) or between the 10 and 30 mg/kg EDO + DOX groups (*p* = 0.3640), whereas the 30 mg/kg EDO + DOX group showed significantly lower MDA levels compared with the 1 mg/kg EDO + DOX group (*p* = 0.0205). To further explore the inflammatory component associated with DOX-induced thyroid injury, tissue levels of pro-inflammatory cytokines were subsequently analyzed.

### 3.4. Tissue TNF-α and IL-6

As depicted in Figure 4B,C, Tissue TNF-α and IL-6 levels differed significantly among the study groups (one-way ANOVA: TNF-α, F(4,30) = 40.03; IL-6, F(4,30) = 43.11; both *p* < 0.0001). The DOX group exhibited significantly higher tissue TNF-α and IL-6 levels than the control group (*p* < 0.0001 for both). In the EDO + DOX treatment groups, TNF-α (*p* = 0.0028, *p* < 0.0001, and *p* < 0.0001) and IL-6 (*p* = 0.0020, *p* < 0.0001, and *p* < 0.0001) levels were significantly reduced relative to the DOX group at doses of 1, 10, and 30 mg/kg, respectively. Within the EDO-treated groups, no significant difference was observed between the 1 and 10 mg/kg doses for TNF-α (*p* = 0.0813) or IL-6 (*p* = 0.2945). By contrast, the 30 mg/kg dose resulted in significantly lower TNF-α (*p* < 0.0001) and IL-6 (*p* = 0.0005) levels than the 1 mg/kg dose, whereas no difference was detected relative to the 10 mg/kg dose (TNF-α *p* = 0.0538; IL-6 *p* = 0.0733) (Figure 4). Finally, to evaluate whether systemic inflammasome activation accompanied these inflammatory changes, circulating levels of NLRP3 inflammasome-related cytokines were measured.

### 3.5. Serum NLRP3, IL-1β, and IL-18

Serum NLRP3, IL-1β, and IL-18 levels differed significantly among the study groups (one-way ANOVA: NLRP3, F(4,30) = 26.65; IL-1β, F(4,30) = 47.07; IL-18, F(4,30) = 60.06; all *p* < 0.0001). Compared with the control group, the DOX group exhibited significantly higher serum levels of NLRP3, IL-1β, and IL-18 (*p* < 0.0001 for all).

Relative to the control group, EDO + DOX treatment at 1 and 10 mg/kg resulted in significantly higher levels of NLRP3 (*p* < 0.0001 and *p* = 0.0084, respectively) and IL-1β (*p* < 0.0001 for both), whereas no significant difference was observed between the control group and the 30 mg/kg EDO + DOX group for NLRP3 (*p* = 0.6192) or IL-1β (*p* = 0.0864). In contrast, all EDO + DOX doses remained significantly higher than the control group for IL-18 (*p* < 0.0256).

Compared with the DOX group, all EDO + DOX treatment groups showed significantly reduced levels of NLRP3 (*p* = 0.0041, *p* < 0.0001, and *p* < 0.0001), IL-1β (*p* = 0.0142, *p* < 0.0001, and *p* < 0.0001), and IL-18 (*p* < 0.0001 for all), at doses of 1, 10, and 30 mg/kg, respectively.

**Figure 5:**
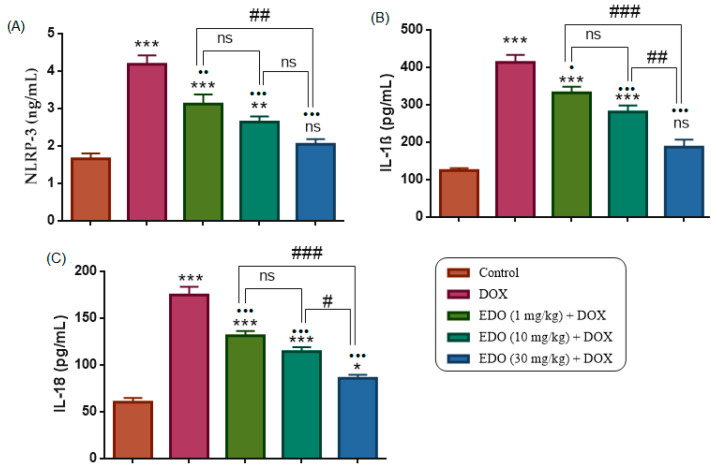
Effects of treatments on (**A**) NLRP3, (**B**) IL-1β, and (**C**) IL-18 levels. Data are presented as mean ± SEM. * *p* < 0.05, ** *p* < 0.01, *** *p* < 0.001 indicates comparisons with the control group; • *p* < 0.05, •• *p* < 0.01, ••• *p* < 0.001 indicates comparisons with the DOX group; # *p* < 0.05, ## *p* < 0.01, ### *p* < 0.001 indicates comparisons among EDO-treated groups. ns denotes non-significant differences.

Within the EDO-treated groups, no significant difference was observed between the 1 and 10 mg/kg doses for NLRP3 (*p* = 0.3999), IL-1β (*p* = 0.2185), or IL-18 (*p* = 0.2353), whereas the 30 mg/kg dose resulted in significantly lower levels compared with the 1 mg/kg dose for NLRP3 (*p* = 0.0033), IL-1β (*p* < 0.0001), and IL-18 (*p* < 0.0001), and compared with the 10 mg/kg dose for IL-1β (*p* = 0.0035) and IL-18 (*p* = 0.0102) (Figure 5)

## 4. Discussion

In the DOX-treated group, serum T3 and T4 levels were markedly reduced, whereas TSH levels were significantly elevated, indicating a hypothyroid state. Consistent with these biochemical findings, scintigraphic imaging revealed decreased thyroid radiopharmaceutical uptake in the DOX group. In parallel, DOX administration was associated with a pronounced increase in oxidative stress and inflammatory markers, as evidenced by elevated levels of MDA, TNF-α, IL-6, NLRP3, IL-1β, and IL-18. In contrast, treatment with EDO, particularly at the 30 mg/kg dose, substantially attenuated these alterations, demonstrating a clear regression of the DOX-induced hypothyroid pattern.

In the previous clinical and experimental studies, thyroid hypo-functioning was observed to develop after chemotherapy. Livesey and Brook [6] showed that the incidence of thyroid dysfunction increased in patients after chemotherapy and that some patients developed secondary hypothyroidism. Another study dealing with advanced-stage Hodgkin’s lymphoma patients reported that 24 of the 54 adults treated with chemotherapy alone developed primary thyroid dysfunction after chemotherapy treatment [29]. Experimental studies on the effects of chemotherapy agents on thyroid function are limited. However, in a remarkable study, levels of maternal and fetal thyroid hormones were evaluated in rats treated with chemotherapy during pregnancy. As in humans, hypothyroidism in animals is diagnosed by measuring total or free T4 level together with plasma TSH concentrations [30]. In a previous study, maternal and fetal serum T4, T3, and thyrotropin (TSH) levels increased in the control group, whereas doxorubicin treatment decreased maternal and fetal T4 and T3 levels but increased TSH levels [31]. In another study, it was found that during pregnancy, injection of doxorubicin caused thyroid agenesis, hypoplasia, and disorder [2].

Plasma TSH, T3, and T4 levels are widely accepted indicators of thyroid functional status. In the present study, DOX administration significantly reduced plasma T3 and T4 levels while increasing TSH levels, suggesting the development of hypothyroid-like alterations. Consistent with these findings, Alhowail [10] demonstrated that repeated DOX exposure markedly decreased circulating total and free T3 and T4 levels, reflecting impaired thyroid hormone synthesis. Similarly, Alotayk et al. [9] reported that high-dose DOX administration resulted in pronounced reductions in T3 and T4 levels accompanied by a significant elevation in TSH, findings consistent with thyroid functional impairment. Aldubayan et al. [11] further showed that DOX significantly reduced T3, T4, FT3, and FT4 levels, with TSH elevation becoming evident under higher cumulative doses or prolonged exposure.

Taken together, these findings suggest that DOX administration may disrupt thyroid hormone homeostasis and trigger a compensatory pituitary TSH response in a dose- and duration-dependent manner. In the present study, treatment with EDO was associated with improvements in thyroid hormone levels and thyroid scintigraphic activity compared with the DOX-treated group. Overall, the present findings suggest that DOX administration is associated with hypothyroid-like alterations in rats, whereas EDO treatment may attenuate these functional changes.

Thyroid scintigraphy is widely used in clinical practice to assess thyroid structure, damage, and functional status. It is among the earliest nuclear medicine techniques used in veterinary medicine [32] and remains a commonly used imaging method. Although early experimental studies primarily focused on cats and dogs, subsequent reports demonstrated that thyroid scintigraphy in rats and mice can yield sufficiently clear images, supporting its applicability to experimental thyroid research in small animals [21]. Among radionuclides, ^99m^Tc pertechnetate is the most frequently used agent and is routinely employed in the scintigraphic evaluation of thyroid disorders [33], with relatively low radiation exposure (~1 mSv). In thyroid scintigraphy, normal thyroid glands typically display a spherical or oval configuration [21,22], and radionuclide uptake intensity reflects thyroid functional activity [34]. Reduced uptake is generally associated with hypothyroidism, whereas increased uptake may indicate hyperthyroidism [22]. In the present study, the DOX-treated group exhibited a pronounced reduction in thyroid functional activity, suggesting that DOX may contribute to thyroid dysfunction at biochemical and functional levels. In contrast, in the EDO-pretreated groups, the observed improvement in serum thyroid hormone concentrations and thyroid functional activity despite doxorubicin administration suggests that EDO attenuates DOX-induced thyroid dysfunction across both biochemical and functional parameters.

Experimental studies have shown that DOX induces oxidative stress and inflammation in various tissues, including the thyroid. Through redox cycling, DOX increases reactive oxygen species (ROS) production, leading to lipid peroxidation and elevated malondialdehyde (MDA) levels [9,10,35,36]. In line with our findings, Alhowail et al. [10] demonstrated that DOX administration significantly increased tissue MDA levels while concurrently reducing circulating T3 and T4 concentrations, supporting the role of oxidative stress in DOX-induced hypothyroidism.

This oxidative damage, in turn, activates stress pathways (e.g., NF-κB via TLR4), promoting the overproduction of pro-inflammatory cytokines such as TNF-α, IL-6, and IL-1β [37,38]. Numerous studies have demonstrated that DOX treatment significantly increases the production of pro-inflammatory cytokines, including TNF-α, IL-6, and IL-1β across various experimental models [9,39,40]. DOX also activates the NLRP3 inflammasome—a key driver of caspase-1–mediated maturation of IL-1β and IL-18—thereby amplifying inflammation [36,41]. Consistent with previous reports, our findings demonstrate that DOX induces marked oxidative stress and inflammatory activation, as evidenced by significant increases in MDA, TNF-α, IL-6, IL-1β, IL-18, and NLRP3 levels. Previous studies and the present findings indicate that DOX promotes lipid peroxidation and inflammation, leading to oxidative stress. Under these conditions, NLRP3 inflammasome activation may impair thyroid follicular cell integrity and suppress thyroid hormone synthesis. Consequently, DOX-induced oxidative and inflammatory stress may contribute to thyroid dysfunction and reduced hormone production. In the present study, EDO reduced these parameters in a dose-dependent manner, with the most pronounced effects observed at the higher dose of 30 mg/kg.

EDO, a potent free-radical scavenger, mitigates DOX-induced thyroid injury. EDO has been shown to eliminate free radicals in various tissues induced by chemotherapy and radiotherapy and to exert an antioxidant effect [13,14]. Mechanistically, EDO activates the Nrf2/HO-1 antioxidant response and inhibits NF-κB signaling [42,43], thereby reducing ROS accumulation and downstream cytokine release. Previous experimental studies have shown that EDO suppresses NLRP3 inflammasome activation in various models [44,45]. Notably, the present study is the first to demonstrate a dose-dependent suppression of NLRP3 in a DOX-induced hypothyroidism model. In the cadmium-induced hypothyroid-like mouse model, serum T3 and T4 levels were not assessed; the protective effects of EDO were demonstrated through attenuation of oxidative stress, inflammatory responses, and associated tissue and neuroinflammatory damage [20]. In experimental autoimmune thyroiditis, EDO reduced inflammation and normalized irregular or elevated T3/T4 levels without affecting TSH [19]. EDO’s antioxidant and anti-inflammatory actions (limiting MDA and cytokine/inflammasome elevations) align with our experimental findings, resulting in improvement of thyroid functional activity and reduction in DOX-induced biochemical alterations. EDO’s antioxidant and anti-inflammatory actions, including suppression of MDA, pro-inflammatory cytokines, and NLRP3 inflammasome activation, may help maintain thyroid functional homeostasis and mitigate DOX-induced hypothyroid-like dysfunction.

In the present study, EDO exerted a dose-related protective effect against DOX-associated thyroid dysfunction. While both 10 and 30 mg/kg doses improved biochemical and inflammatory parameters, the incremental benefit of dose escalation was outcome-dependent. Notably, the 30 mg/kg dose produced greater improvement in functional scintigraphic recovery of thyroid radiotracer uptake, whereas the recovery of circulating thyroid hormones and several systemic oxidative and inflammatory markers appeared to plateau beyond 10 mg/kg. This heterogeneous dose–response pattern may reflect the dose-dependent radical-scavenging activity of edaravone and the distinct exposure thresholds required for downstream pathway modulation [13]. Consistent with this interpretation, previous studies have demonstrated pharmacological efficacy of edaravone within the 10–40 mg/kg range in rodent models, with more pronounced protective effects at higher doses [19]. Taken together, these findings suggest that 10 mg/kg may represent an effective intermediate dose for attenuating systemic oxidative and inflammatory responses, whereas 30 mg/kg may provide additional functional benefit for thyroid activity, particularly in imaging-based functional assessments.

Importantly, Edaravone was administered intraperitoneally in the present study, systemic absorption primarily occurs through the peritoneal vascular bed rather than the intestinal lumen; therefore, the contribution of intestinal microbiota to the absorption phase is expected to be minimal [46,47,48].

Anthracycline-based chemotherapy has been associated with thyroid dysfunction in clinical settings, particularly hypothyroidism. For example, thyroid dysfunction has been reported in approximately 14.8% of breast cancer patients receiving anthracycline-containing regimens, with some requiring levothyroxine therapy [49]. Our findings suggest that doxorubicin-associated thyroid dysfunction may be mediated, at least in part, by oxidative and inflammatory mechanisms. In this context, edaravone, a potent free radical scavenger, may represent a potential protective strategy. Importantly, previous studies have suggested that edaravone does not interfere with the antitumor activity of anthracyclines, supporting its potential use as an adjunctive protective agent [50].

Edaravone is clinically approved for acute ischemic stroke and amyotrophic lateral sclerosis (ALS) at 60 mg/day and has a well-established safety profile [51]. Experimental studies have also demonstrated good tolerability in rodents across a dose range of 1–40 mg/kg without major adverse effects [19,52]. Consistent with these reports, the present study demonstrated that edaravone attenuated DOX-associated hypothyroid-like alterations across all tested doses. Notably, protective effects were observed even at the lowest dose (1 mg/kg), suggesting that edaravone may exert biological activity at relatively low doses, while the protective effects were more pronounced at 10 and 30 mg/kg. Nevertheless, further clinical studies are required to confirm these findings.

## 5. Conclusion

Our findings demonstrate that doxorubicin markedly impairs thyroid function, evidenced by reduced radiotracer uptake, the suppression of T3 and T4, elevation in TSH, and the pronounced activation of oxidative stress and inflammatory pathways. Importantly, edaravone pretreatment significantly attenuated these pathological alterations in a dose-dependent manner, preserving thyroid uptake on scintigraphy, attenuating hormonal disturbances, and suppressing NLRP3-associated inflammatory responses. These results provide novel evidence that edaravone exerts a protective effect against chemotherapy-induced thyroid dysfunction.

### Limitation

The present study has several limitations. First, histopathological evaluation of thyroid tissue was not performed. Although scintigraphic imaging and biochemical analyses provided functional evidence of thyroid dysfunction, histological confirmation of follicular structural alterations would further strengthen the findings. Second, an a priori power analysis was not conducted, and the sample size (n = 7 per group) was determined based on ethical considerations and consistency with similar preclinical studies. Therefore, future studies with larger sample sizes and predefined power calculations are needed to further validate these results.

## Data Availability

The data that support the findings of this study are not publicly available due to ethical reasons but are available from the corresponding author upon request.
